# Multilocus sequence typing (MLST), *por*A and *fla*A typing of *Campylobacter jejuni* isolated from cats attending a veterinary clinic

**DOI:** 10.1186/s13104-019-4107-5

**Published:** 2019-02-04

**Authors:** Vathsala Mohan, Ihab Habib

**Affiliations:** 1 Institute of Veterinary, Animal and Biomedical Sciences, Massey University, Palmerston North, New Zealand; 2 School of Veterinary Medicine, Murdoch University, Perth, Western Australia Australia; 3 High Institute of Public Health, Alexandria University, Alexandria, Egypt

**Keywords:** *Campylobacter*, Pets, Cats, MLST, Genotyping, *por*A, *fla*A typing

## Abstract

**Objective:**

Campylobacter is a major cause of gastroenteritis in humans and pet ownership is a risk factor for infection. To study the occurrence, species distribution and sequence-based types of *Campylobacter* spp. in pet cats, 82 faecal samples were collected from cats in New Zealand. The PCR positive samples of *Campylobacter jejuni* were characterized by multilocus sequence typing (MLST), major outer membrane protein gene (*por*A) and flagellin A gene (*fla*A) sequence typing.

**Results:**

Seven faecal samples were tested positive for *Campylobacter* spp. (9%, or 4–17% at 95% confidence interval), of which six were identified as *C. jejuni*, and one was *C. upsaliensis*. The six *C. jejuni* isolates were characterised by MLST; four belonged to ST-45 clonal complex and two of the isolates could not be typed. Two *fla*A-SVR types were identified: three samples were *fla*A-SVR type 8 and one belonged to 239. By combining all data, three isolates were indistinguishable with allelic combinations of ST-45, *fla*A-SVR 8, *por*A 44, although no epidemiological connection between these isolates could be established. To conclude, healthy cats can carry *C. jejuni*, whose detected genetic diversity is limited. The isolated sequence type ST-45 is frequently reported in human illnesses.

## Introduction

*Campylobacter* spp. is the major cause of bacterial gastroenteritis in many countries, including New Zealand [1–3]. Most human infections are due to *Campylobacter jejuni* and *C. coli*, and other *Campylobacter* spp. can be commonly isolated from clinical cases of campylobacteriosis [4–7]. Risk factors of human campylobacteriosis include ingestion of untreated water, undercooked meat particularly poultry meat, raw milk, cross-contamination of foods, and contact with animals including pets and wild birds [8–10]. Different foodborne transmission pathways have been extensively studied to reveal the epidemiology of *C. jejuni* in New Zealand [3, 11, 12], however, animal contacts and transmission of *Campylobacter* through pets has been studied less extensively. A number of studies from other countries have indicated that pets, in particular, young animals, can transmit these pathogens to their owners and that, household pets pose a significant risk to their owners which was reviewed by Pintar, Christidis [13]. Campylobacteriosis incidence in New Zealand was reported to be 396 cases per 100,000 population in 2003 [14] which was the highest rate compared to other developed countries worldwide. The incidence dropped to 157 cases per 100,000 in 2008 which remained stable until 2012 [15]. However, the current incidence is reported to be 1.5 to 3 times higher than countries including Australia, England and Wales, and several other Scandinavian countries with a likelihood of more than 1% of New Zealanders acquiring the disease each year [16, 17]. Moreover, in 2016, a total of 964 waterborne campylobacteriosis cases were reported, that included 941 cases from the Hawkes Bay region District Health Board (DHB) and 23 cases from other DHBs and this outbreak was reported to be a largest waterborne outbreak in New Zealand [18].

Although New Zealand has high numbers of human campylobacteriosis clinical cases as against other developed countries, there is still only a very limited data available on the prevalence of *C. jejuni* in pets in NZ [19] and comparatively more data is available for dogs than cats [20, 21].

The estimates of *C. jejuni* prevalence in cats varies widely between countries, ranging from 1.3 to 16.8% [22–28] with higher prevalence in kittens (14.3%) and diarrhoeal cats (23.8%) [29]. Although kittens may thus pose a larger risk of *Campylobacter* carriage than adult animals, owners are more typically exposed to adult animals than to kittens. Thus, we concentrated on animals more than 6 months of age or older.

In order to evaluate the role of cats in transmitting *Campylobacter* species to humans, feline samples must be typed by means of methods also applied to human samples. For this, we selected multilocus sequence typing (MLST) [30]. By using MLST, isolates are characterized by partial sequencing of seven housekeeping genes whose allele combination groups strains into sequence types (ST) which in turn can be grouped into clonal complexes (CC) [31].To add additional discrimination power, we combined this method with typing of *fla*A and *por*A sequences [32].

## Main text

### Methods

#### Collection of faecal material

Faecal material was collected from 74 cats attending the Massey University Small Animal Veterinary Clinic for routine procedures such as vaccination, de-worming and neutering and four cats (adults) belonging to staff and students of Massey University and a commercial cattery (Table 1).

**Table 1:** Faecal material collected from cats from March 2008 to July 2010

Kittens (< 6 months)	Young adults (9 to < 24 months)			Adults (> 24 months)				
Massey University clinic healthy	Massey University clinic healthy	Massey University clinic from SPCA healthy	Wet pets shop	Received medication for fungal infection^a^	Diarrhoea^a^	Healthy household^a^	Cattery	Total
2	69	3	2	2	1	2	1	82

^a^Massey University staff and student

Sample collection continued from March 2008 through July 2010. A total of 82 faecal samples were collected, the majority of which (n = 69) came from cats under 2 years of age.

#### Bacterial isolation and DNA preparation

Bacterial isolation and DNA preparation were carried as described elsewhere [20]. Faecal material was collected in transport media and directly streaked onto modified charcoal cefoperazone-deoxycholate plates (mCCDA) (Fort Richards, Auckland) and also directly inoculated into Bolton’s enrichment broth; both were incubated for 48 h at 42 °C under microaerophilic conditions. *Campylobacter* presumptive pure colonies were tested for oxidase reduction (oxidase strips, Fort Richards, Auckland) and positive isolates were transferred into 1 mL of 2% (weight/volume) Chelex solution in distilled water and boiled for 10 min. Following centrifugation, at 13,000 rpm for 10 min, the supernatants were directly used as a template for PCR reactions and MLST typing.

#### Characterisation of *C. jejuni* by PCR

All samples were subjected to a PCR amplifying the 16s rRNA gene to confirm the genus *Campylobacter* using published primers (forward: 5′ GGATGACACTTTTCGGAGC 3′; reverse: 3′ CATTGTAGCACGTGTGTC 3′) [21]. Isolates confirmed by PCR to belong to the genus *Campylobacter* were further speciated as *C. jejuni* based on amplification of the membrane-associated protein A (*map*A) gene using published primers (forward: 5′ CTTGGCTTGAAATTTGCTTG 3′; reverse: 3′ GCTTGGTGCGGATTGTAAA 5′) [32]. *C. upsaliensis* primers, PCR reaction and conditions were adapted from a previously published report [33].

#### Multilocus sequence typing, *fla*A typing and *por*A typing

All the *C. jejuni* positive isolates were subjected to MLST characterization as described and the primers used were adapted from previous reports on MLST characterization [32, 34]. The PCR conditions and primers for *fla*A and *por*A typing were obtained from the PubMLST database and their allelic numbers were assigned by referring to the same *Campylobacter* PubMLST database.

#### Data analysis

Descriptive analysis of the data was carried out using epiR [35] statistical package, with the calculation of exact binomial 95% confidence interval.

### Results and discussion

Of 82 faecal samples obtained from in this study, 7 were positive for *Campylobacter* sp. based on the 16S rRNA PCR, of which 6 were positive for *C. jejuni*, based on the sequence of their *mapA* gene (the seventh isolate was determined as *C. upsaliensis*). This corresponds to an estimated carriage rate of 9% for *Campylobacter* spp. (95% CI, 4–17%) and of 7% (95% CI 3 to 15%) for *C. jejuni*, which is within the range of feline Campylobacter carriage reported from other studies. The prevalence of *Campylobacter* species in cats has been consolidated in few studies that had reported the *Campylobacter* spp. to range from 0.8 to 78%; *C. jejuni* from 1 to 31%; *C. upsaliensis* from 5 to 66% and *C. lari* from 0–4% [26, 36]. Our prevalence in kittens is lower than the reported prevalence of *C. jejuni* in kittens (14.3%) and cats with diarrhoea (23.8%) [29]. The authors of the latter work commented on a striking difference between cats below the age of 1 year and adult animals, as they found only *C. jejuni*-positive animals among the positive kittens. Similarly, in our study, there were two kitten samples from the entire pool of 82 samples and both were positive for *C. jejuni*. Other studies also reported higher prevalence in young cats (eg. 30% in cats under 2 years of age compared to 3% in adult animals [28], and some studies reported *C. upsaliensis* or *C. helveticus* to be prevalent [37]. Nevertheless, studies suggest that age was not a risk factor Campylobacter carriage in cats and dogs [22, 37]. In our study, most (n = 69) of the sampled cats were younger than 2 years. The majority of the sampled animals were reared in households and a number of cats were permanently kept indoors, which may have caused a lower prevalence due to lack of exposure opportunities. Nevertheless, our results show that even under these conditions healthy kittens, young but adult cats can harbour *C. jejuni* and can act as possible sources of this pathogen to their owners.

Gastrointestinal health has been indicated as a risk factor for isolating Campylobacters from pets, and intestinal symptoms have been identified as a risk factor for the isolation of *C. jejuni* and *C. upsaliensis* [38]. The single cat with diarrhoea of our study was positive for *C. jejuni*, however, the owner stated that it was an outdoor cat that only returned home during the night. It has been observed that diverse *Campylobacter* spp. may be opportunistic colonizers in diarrhoeal pets [39]; similarly, evidence indicates humans with diarrhoea can carry up to 5 species of Campylobacter or related organisms [7]. Intensive housing and open drains were reported to increase the carriage rate by 2 times in cats [40]. A number of studies suggested the absence of a correlation between the presence of diarrhoea or intestinal disorders and isolation of *Campylobacter* in cats [22, 37, 38, 41–43].

We could not establish whether the presence of the *C. jejuni* was the cause or consequence of the diarrhoea, but treatment with antibiotics (details of the antibiotics not known) was reported to have resolved the problem. Two other adult cats from the Massey University staff were receiving antifungal or antimicrobial treatment for fungal infection, therefore we expected that these samples to be negative for *C. jejuni* and so they were.

The six *C. jejuni* isolates obtained were subjected to MLST typing, but only four *C. jejuni* isolates could be successfully typed. For two isolates even after repeated attempts with superior polymerase enzyme and optimised magnesium chloride concentrations (2.5 mM) not all alleles could be amplified, which we consider was due to lack of primer consensus, in particular near to the 3′-end of the primer, which would have hampered correct annealing and amplification [44]. Thus, these two isolates remained with incomplete profile and could not be evaluated by MLST. However, alternate primers can be designed to type these difficult alleles. Due to limited resources and small number of isolates, further attempts with alternate primers and modified conditions were not carried out at that time. Nevertheless, we acknowledge that these factors should be addressed. The four MLST typed samples were assigned to ST-45 (n = 3) one of them was from the adult cat with diarrhoea and ST-583 (n = 1), both belonging to Clonal Complex (CC) 45. In an attempt to increase discrimination their *fla*A and *porA* alleles were also determined. This showed the three ST45 isolates from kittens to be indistinguishable, with *flaA* allele 8 and *por*A allele 44, while the ST-583 isolate produced *fla*A allele 239 and *por*A allele 73 which was from the adult cat with diarrhoea (Table 2). Two samples could not be typed after repeated attempts. There are studies on multilocus sequence typing for dog *C. jejuni* isolates [21, 45–47], while few studies have carried out using random amplified polymorphism (RAPD) analysis [23]. One recent study reported that in cats two isolates belonging to ST-696 and one ST-48 were detected from cats’ rectal samples [19] from the same geographical area as that of our study. While interrogating our data for the occurrence of these ST across different sources with the mEpiLab database (IVABS, The Hopkirk Institute, Massey University, NZ), it was evident that these STs are frequently encountered in human clinical cases in that geographical area. Even one of the isolates for which a complete MLST profile could not be established produced a combined *fla*A-239, *por*A-73 genotype, suggesting it might have been similar to the ST-583 (CC45) and another isolate without a complete MLST profile produced a *por*A-73. Such a low level of diversity in feline isolates was unexpected and could suggest a link between the animals producing these isolates based on the date of isolation, but the geographical location of their homes was not similar.

**Table 2:** *C. jejuni* genotypes from 6 feline faecal samples

Source	No. of isolates	ST	CC	*asp*A	*gln*A	*glt*A	*gly*A	*pgm*	*tkt*	*unc*A	*fla*A	*por*A	Age (months)	Health status
Massey University Vet. Clinic	1	45	45	4	7	10	4	1	7	1	8	44	6	Healthy
Massey University Vet. Clinic	1	45	45	4	7	10	4	1	7	1	8	44	5	Healthy
Massey University Vet. Clinic	1	45	45	4	7	10	4	1	7	1	8	44	< 24	Healthy
Massey University staff	1	583	45	4	7	10	4	42	51	1	239	73	> 24	Diarrhoea
Massey University staff	1	U/A	x	x	x	x	x	x	x	x	x	73	< 24	Healthy
Wet pets shop	1	U/A	x	x	x	x	x	x	x	x	239	73	< 24	Healthy

This table describes the source, frequency of sequence type (ST), clonal complex (CC), the MLST allelic profile, *fla*A and *por*A alleles, age and health status

x: allele that could not be sequence typed, U/A: unassigned

### Conclusion

In conclusion, this study has quantified the carriage rate of *Campylobacter* spp. in cats in an urban area of New Zealand. Although there is a general conception that healthy pets do not carry *C. jejuni*, our results have shown that healthy cats did carry zoonotic *C. jejuni* (ST-45 and ST-583) populations which may pose a potential risk for their owners. Besides, cats themselves may suffer from inflammatory bowel disease [48].

Furthermore, the *por*A and *fla*A alleles found in cats in this study were identical to those from human campylobacteriosis recorded in the PubMLST database which speculates that pets may represent a risk factor for human infection and that this risk may vary on the personal hygiene practices followed by the pet owners.

The recovery of zoonotic genotypes from cats’ faecal material provides evidence for *C. jejuni’s* survival ability outside the host where the samples were collected from faecal materials. The samples were collected after the animals defecated and in all of the occasions the animals were allowed to wander outside the out-patient ward for them to be able to defecate before any of the clinical procedures were conducted. This further emphasizes the need for following hygienic procedures and practices for the benefit of the cat owners. Our study along with other previous studies emphasizes that outdoor cats have to be dealt with care when they mingle with family members as they have higher odds of carrying Campylobacters. We also suggest investigating for sharing of food between the owners and their pet cats could provide insights into the overlap of genotypes between humans and cats. Different sampling strategies including swab sampling from the rectum, accounting for risk factors including exposures to different types of food sources (processed food sources, home-prepared foods and wild hunting) housing patterns of cats (indoor or outdoor), age and health conditions and medications have to be taken into account as all these factors influence the probability of detecting the pathogen, the direction of transmission and the precision of the inference.

## Limitations

Although New Zealand has the highest campylobacteriosis incidence rates which are reported to be 1.5 to 3 times higher than other developed countries with a predicted likelihood of more than 1% of New Zealanders being affected by campylobacteriosis every year, this study provides information about an additional potential source for it. We acknowledge that there are few limitations to this study. The sample size was small (82) and specimens were collected from 4 sources: Massey University Small Animal Veterinary Clinic, University staff, a private veterinary clinic and a commercial cattery collected during from March 2008 to July 2010. Because the number of samples and the sampling pattern were not consistent, these samples were not used to estimate the prevalence of *C. jejuni* in cats, rather, the positive samples were used for the characterisation and population differentiation of *C. jejuni* in cats. However, this study certainly provides an opening to consider cats as potential reservoirs of *C. jejuni* and emphasises that the cat owners have to be mindful about their hygienic practices although the sample may not represent the entire cat population.
